# Validity and reliability of the Traditional Chinese version of the Multidimensional Fatigue Inventory in general population

**DOI:** 10.1371/journal.pone.0189850

**Published:** 2018-05-10

**Authors:** Li-Ling Chuang, Yu-Fen Chuang, Miao-Ju Hsu, Ying-Zu Huang, Alice M. K. Wong, Ya-Ju Chang

**Affiliations:** 1 School of Physical Therapy and Graduate Institute of Rehabilitation Science, College of Medicine, and Healthy Ageing Research Center, Chang Gung University, Taoyuan, Taiwan; 2 Healthy Ageing Research Center, Chang Gung University, Taoyuan, Taiwan; 3 Department of Physical Medicine and Rehabilitation, Chang Gung Memorial Hospital, Linkou, Taoyuan, Taiwan; 4 Department of Physical Therapy, College of Health Science, Kaohsiung Medical University, Kaohsiung, Taiwan; 5 Department of Physical Medicine and Rehabilitation, Kaohsiung Medical University Hospital, Kaohsiung, Taiwan; 6 Department of Neurology, Chang Gung Memorial Hospital, Linkou, Taoyuan, Taiwan; 7 Institute of Cognitive Neuroscience, National Central University, Taoyuan, Taiwan; 8 School of Medicine, College of Medicine, Chang Gung University, Taoyuan, Taiwan; 9 Neuroscience Research Center, Chang Gung Memorial Hospital, Linkou, Taoyuan, Taiwan; PLoS, UNITED STATES

## Abstract

**Background:**

Fatigue is a common symptom in the general population and has a substantial effect on individuals’ quality of life. The Multidimensional Fatigue Inventory (MFI) has been widely used to quantify the impact of fatigue, but no Traditional Chinese translation has yet been validated. The goal of this study was to translate the MFI from English into Traditional Chinese (‘the MFI-TC’) and subsequently to examine its validity and reliability.

**Methods:**

The study recruited a convenience sample of 123 people from various age groups in Taiwan. The MFI was examined using a two-step process: (1) translation and back-translation of the instrument; and (2) examination of construct validity, convergent validity, internal consistency, test-retest reliability, and measurement error. The validity and reliability of the MFI-TC were assessed by factor analysis, Spearman rho correlation coefficient, Cronbach’s alpha coefficient, intraclass correlation coefficient (ICC), minimal detectable change (MDC), and Bland-Altman analysis. All participants completed the Short-Form-36 Health Survey Taiwan Form (SF-36-T) and the Chinese version of the Pittsburgh Sleep Quality Index (PSQI) concurrently to test the convergent validity of the MFI-TC. Test-retest reliability was assessed by readministration of the MFI-TC after a 1-week interval.

**Results:**

Factor analysis confirmed the four dimensions of fatigue: general/physical fatigue, reduced activity, reduced motivation, and mental fatigue. A four-factor model was extracted, combining general fatigue and physical fatigue as one factor. The results demonstrated moderate convergent validity when correlating fatigue (MFI-TC) with quality of life (SF-36-T) and sleep disturbances (PSQI) (Spearman's rho = 0.68 and 0.47, respectively). Cronbach’s alpha for the MFI-TC total scale and subscales ranged from 0.73 (mental fatigue subscale) to 0.92 (MFI-TC total scale). ICCs ranged from 0.85 (reduced motivation) to 0.94 (MFI-TC total scale), and the MDC ranged from 2.33 points (mental fatigue) to 9.5 points (MFI-TC total scale). The Bland-Altman analyses showed no significant systematic bias between the repeated assessments.

**Conclusions:**

The results support the use of the Traditional Chinese version of the MFI as a comprehensive instrument for measuring specific aspects of fatigue. Clinicians and researchers should consider interpreting general fatigue and physical fatigue as one subscale when measuring fatigue in Traditional Chinese-speaking populations.

## Introduction

Fatigue is a common and frequently distressing symptom in the general population [1–3] and among patient populations such as cancer, rheumatoid arthritis, and stroke [4–7]. It has been shown to have a substantial impact on individuals’ quality of life [8]. Fatigue in healthy people is usually temporary and is conceptualized as a consequence of physical or mental exertion [7,9]. Most individuals experience fatigue after inadequate sleep or rest, after the exertion of physical activity, after mental effort, or when they lack the motivation to initiate activities.

Defining fatigue is difficult because it is a complex and multidimensional concept, comprising physiological, emotional, and mental aspects [10]. Aaronson and colleagues [11] defined fatigue as “the awareness of a decreased capacity for physical and/or mental activity due to an imbalance in the availability, utilization, and/or restoration of resources needed to perform activity.” Physiological fatigue is generally caused by excessive energy consumption, depletion of essential substrates of physiological functioning, and/or a diminished ability to contract muscles. Psychological fatigue is defined as “a state of weariness related to reduced motivation, prolonged mental activity, or boredom that occurs in situations such as chronic stress, anxiety or depression [12].”

Comparisons between fatigue and similar concepts from well-known questionnaires have not been done in empirical studies. However, the content of the vitality subscale of the Short Form-36 Health Survey (SF-36) is closely related to general fatigue [13]. Sleep disturbances have also been shown to induce self-reported fatigue in patients with rheumatoid arthritis [14] and traumatic brain injury [15] and in spouse caregivers of cancer patients [16]. Fatigue due to sleep deprivation may place individuals at increased risk for injuries, degraded health, and impaired physical and mental performance [17].

Because of its high prevalence and increasingly acknowledged negative effect on individuals’ well-being, fatigue has become an important topic of research. Such research is important for the development of cultural adaptations of measures of fatigue, for early detection of fatigue severity, and for the interpretation of the levels of fatigue found in general and patient populations. A comprehensive instrument with good psychometric properties for measuring specific aspects of fatigue is needed to identify individuals with fatigue.

Instruments available to assess fatigue can be divided into one-dimensional instruments and multidimensional instruments. Clinicians usually evaluate fatigue severity of the patients based on a self-reporting scale, such as the Numerical Rating Scale [18], Visual Analogue Scale [19], and the Brief Fatigue Inventory [20]. However, these scales take a one-dimensional approach to the concept of fatigue. In so doing, they cannot comprehensively represent the multidimensional nature of fatigue in the general population, and they impose several limitations on research results and subsequent interpretations [21]. In contrast, multidimensional measures of fatigue allow a comprehensive assessment of subjects’ perceptions and a detailed picture of subjects’ fatigue in both physical and mental dimensions. Multidimensional instruments permit the exploration of the structure of a patient’s perception of fatigue [21].

A good example of the multidimensional approach of fatigue instrument is the Multidimensional Fatigue Inventory (MFI). The MFI is based on the definition of fatigue given above and is an easy-to-administer scale [22]. The original MFI assesses 5 dimensions of fatigue: general fatigue, physical fatigue, mental fatigue, reduced activity, and reduced motivation [23]. The MFI has been widely used to measure fatigue, not only in the general population [24–26], but also in cancer patients [22,27,28], individuals with chronic fatigue syndrome [22], fibromyalgia patients [29], and patients with Parkinson’s disease [30]. A previous study evaluated the internal consistency of the MFI and found that the Cronbach’s α coefficient of the subscales of the MFI ranged from 0.53–0.93. Construct and convergent validity was established via correlations with a Visual Analogue Scale measuring fatigue (r ranges = 0.22–0.78) [22]. The MFI is a psychometrically appropriate instrument for the evaluation of fatigue in patient populations, with high internal consistency and high validity [22,31]. Despite the wide acceptance of the MFI worldwide, translation and cross-cultural adaptation to a Traditional Chinese version has not previously been reported. Given the high prevalence rate of fatigue in Taiwan [32] and the importance of having questionnaires in the native language of the subjects, there is an urgent need to generate a cultural adaptation of fatigue measure that is appropriate for the general population of Taiwan. Development of a Traditional Chinese version of the MFI will allow better standardizing of clinical practice for early detection of fatigue severity and allow health professionals treating specific aspects of fatigue symptom with a globally accepted outcome measure. It is important to investigate the reliability and validity of the Traditional Chinese version of the MFI (MFI-TC) in Taiwanese population to assess whether the MFI-TC could be a complementary screening tool in population with fatigue symptom.

Factors that may influence individuals' perception of fatigue such as ethics and values result in the challenges of fatigue measure [21]. In addition, cultural issues and language peculiarities can affect the process of fatigue assessment. The MFI has been translated into French, Hindi, Polish, Simple Chinese, Swedish, and Spanish [27–29,33–36] to quantify fatigue severity, but no Traditional Chinese translation has yet been validated to evaluate fatigue status in the general population of Taiwan. In addition, although the reliability and validity of the MFI in cancer patients were described in previous studies by Smets [22,31], no psychometric evaluation of the MFI in a general population has been reported. The need for reference data from the general population of Taiwan and for psychometric testing of the MFI-TC was the main reason for this study. Therefore, the aims of this study were (a) to translate the English version of the MFI into Traditional Chinese, (b) to examine the psychometric properties (construct validity, convergent validity, internal consistency, test-retest reliability, and measurement error) of the MFI-TC to establish its utility in Taiwanese clinical and research practice.

## Materials and methods

### Participants

The required sample size was estimated to be at least 100 by the G-Power program, with an alpha of 0.05, a power of 0.80 and a moderate correlation of 0.5. A preferable sample of more than 100 observations is required for factor analysis [37].With a desired ratio of 5 observations per variable, a sample of more than 100 observations is required for the 20 items of the MFI-TC. This study adopted a cross-sectional research method with convenient sampling. Some principles for sampling were as follows: First, our survey targeted relevant people in one university and one association. Second, institutions willing to cooperate with the researchers were eligible for this study. Furthermore, all potential subjects were requested to complete a self-reported questionnaire to assess their general health conditions, including physical and psychological health for eligible screening. Perceived physical and mental health was measured by the following single question, respectively: “How would you rate your overall physical/mental health at the present time?” The answer was a dichotomous “healthy” or “unhealthy.” Among the eligible healthy subjects, 150 citizens agreed to take part voluntarily in the study and 123 of the participants (response rate = 82%) completed all of the questionnaires on both occasions, which were usable for analysis. The participants included students, faculty, staff, and family members from Chang Gung University (CGU) and Young Women’s Christian Association of Taiwan (YWCA) in two metropolitan cities in Taiwan (one in Taiyuan, one in Taipei). The inclusion criteria for participants were: (1) aged 20 years or above; (2) volunteers in good physical, cognitive and mental health, per self-report; and (3) capable of speaking and reading Chinese. The exclusion criteria were: (1) participation in any experimental or drug studies during the study period; (2) treatment of fatigue; and (3) severe cognitive deficits. The study was conducted according to the Declaration of Helsinki and was approved by the Institutional Review Board of Chang Gung Memorial Hospital (IRB serial number: 104-0704B). All participants provided written informed consent and were informed of the study’s purpose, the process, and their right to withdraw from the study at any time.

### Procedures

The study was carried out from March 2015 to August 2015. The number of clinical trial registration is NCT02596139. The development of a Traditional Chinese version of the MFI was followed the internationally accepted guideline for the cultural adaptation of a self-reporting questionnaire by Beaton et al [38]. The MFI was examined in a two-step process: (1) translation and back translation; and (2) testing for validity and reliability of the formal MFI-TC. The English version of the MFI was initially translated into the first Traditional Chinese version by one bilingual translator after obtaining approval from the original developer. Eight experts in the areas of physical therapy, sports medicine, questionnaire design, and fatigue were asked to rate the 20 items’ translation accuracy, relevance, and expression fluency on a 5 point-Likert scale (1 = very inappropriate, 5 = very appropriate). This process ensured that the words used in the Traditional Chinese translation meant the same thing as the words used in English to describe aspects of fatigue. The content validity index was used to estimate the validity of the items [39] and quantify the extent of agreement between the experts [40]. The terminology and content of the first MFI-TC were revised based on the experts’ opinions and cultural concerns. An authorized native US-English-speaking translator was asked to blindly back-translate from the modified Traditional Chinese version into the English version to check its equivalence to the original English version. The use of bilinguals and pretesting was a complement to the back-translation technique for assessing and validating a translation [41]. The administration of the questionnaire instrument in both the original English and the modified MFI-TC version to bilingual participants was used to compare their responses to the two versions. Sixty-six bilingual adults were recruited to complete the original English MFI first and the modified MFI-TC version 30 minutes later to test the reliability and concurrent validity of the translation. The translation reliability and concurrent validity of the modified MFI-TC version were assessed by intra-class correlation coefficient (ICC) and Spearman correlation coefficient between the scores of the original English MFI and the modified MFI-TC. The results showed that there were good reliability and concurrent validity of the translation with the value of ICC for total fatigue score: 0.91; and Spearman correlation coefficient (ρ): 0.86 (S1 Table). This ensured that a consistency in the content between the source and target versions of the MFI. The possible limitation of short time intervals (having the same participants complete the two versions 30 minutes apart from one another) is a greater potential for carry-over or recall effects, which will likely overestimate the reliability of the instrument. Based on the results of reliability, validity, and the back translation, the formal Traditional Chinese version of the MFI (MFI-TC) was generated. It should be noted that it does not address the construct validity and test-retest reliability that are critical to describe a successful cross-cultural adaptation.

For determining test-retest reliability, the MFI-TC was assessed twice with a 1-week interval to reduce the memory effect of the first assessment, and at the same time of day to minimize diurnal variation in fatigue. The participants were asked to complete the questionnaires on 2 separate occasions. The initial questionnaires were completed at the time of recruitment and the second set of the MFI-TC was provided in a reply-paid envelope, and participants were asked to complete questionnaire at home and return it with a week apart. Convergent validity of the fatigue measures with the Short-Form-36 Health Survey Taiwan Form (SF-36-T) [21,42] and with the Chinese version of the PSQI [43] was commonly tested in previous validation of the fatigue study. We visited participants in classrooms and offices at CGU and YWCA, explained the study purpose and procedure, and received informed consent from them. Those who agreed to participate in the study completed the MFI-TC, SF-36-T, and the Chinese version of the Pittsburgh Sleep Quality Index (PSQI) questionnaires at that time and received retest of the MFI-TC 1 week later.

### Outcome measures

#### The MFI-TC

Fatigue was measured by the MFI-TC, consisting of a 20-item self-report scale originally designed to evaluate 5 dimensions of fatigue (general fatigue, physical fatigue, reduced activity, reduced motivation, and mental fatigue) [22]. Subjects used a Likert scale ranging from 1 (strongly agree) to 5 (strongly disagree) to indicate how aptly certain statements regarding fatigue represented their experiences. Ten positively phrased items (item 2, 5, 9, 10, 13, 14, 16, 17, 18, 19) were reverse-scored before adding up scores. The total score obtained simply by adding 20-item scores together (i.e., 20–100), with higher scores indicating more fatigue.

#### The SF-36-T

We used the well-validated, self-administered SF-36-T questionnaire to document health-related quality of life. The SF-36-T has 36 questions examining 8 dimensions of the participant’s general health, including physical functioning, physical roles, bodily pain, general health, vitality, social functioning, emotional roles, and mental health. Its creators have also developed algorithms to calculate two psychometrically based summary measures: a physical and a mental component summary score [44]. A high total score means a good quality of life. The Cronbach’s alpha of the SF-36-T is between 0.84–0.88 [45]. It also has good construct validity and content validity [46,47].

#### The Chinese version of the PSQI

The severity of sleep disturbance was evaluated by the Chinese version of the PSQI, a widely used, self-report questionnaire that assesses sleep quality during the previous month [48,49]. The Chinese version of the PSQI consists of 19 self-rated questions and 5 questions rated by the bed partner or roommate. The 19 items are grouped into 7 component scores, each weighted equally on a 0–3 scale. The 7 components are subjective sleep quality, sleep latency, sleep duration, habitual sleep efficiency, sleep disturbances, use of sleeping medications, and daytime dysfunction. The component scores are summed to yield a global PSQI score, which has a range of 0–21. Higher scores indicate worse sleep quality [48]. The validity of the Chinese version of the PSQI has been supported in cancer patients [50].

### Data analysis

All data of the MFI-TC, SF-36, and PSQI are provided in the supplementary materials (S1 Dataset). The construct validity, criterion validity, internal consistency, test-retest reliability, and measurement error of the formal MFI-TC were computed. The construct validity of the MFI-TC was tested by factor analysis (exploratory factor analysis) (IBM SPSS Statistics version 20, IBM Corp, Armonk, NY). A Principal Components Analysis (PCA) was used to extract factors. As correlations between factors were expected, the obtained factors were rotated obliquely using the direct oblimin procedure. A minimal eigenvalue of 1 was specified as an extraction criterion and the criterion for factor loading was set at >0.40. The existing names of the MFI subscales were used to label the extracted factors [22]. Data from the first measurement were used for analysis.

The convergent validity of the MFI-TC was examined by calculating the Spearman correlation coefficients between the scores of the MFI-TC and the SF-36-T and between the scores of the MFI-TC and the PSQI. To offset the effect of multiple correlations and avoid the increased chance of a type I error, the statistical significance level of correlation coefficients was adjusted by Bonferroni’s correction [51]. The significance level of coefficients is indicated only when they reach the 0.001 criterion. The following cutoffs were used to define the magnitude of the correlation coefficients: <0.25, low correlation; 0.25 to 0.5, fair correlation; 0.5 to 0.75, moderate-to-good correlation; and >0.75, good-to-excellent correlation [51].

The examination of reliability of the MFI-TC included tests for internal consistency, test-retest reliability, and measurement error. Internal consistency is the degree of the interrelatedness among scale items measuring a homogeneous construct or characteristic. Internal consistency of the MFI-TC was assessed by calculating Cronbach’s alpha coefficient for the total scale and for all subscales separately at the first measurement. A Cronbach’s alpha coefficient >0.7 implies good internal consistency of a scale [51]. Test-retest reliability over a 1-week interval was determined by computing the intra-class correlation coefficient (ICC) for both the total and subscale scores on the MFI-TC at the initial and subsequent administrations using a 2-way mixed-effect model with an agreement coefficient [52]. ICCs that exceed 0.75 indicate good reliability [51]. We used the standard error of measurement (SEM), the minimal detectable change (MDC), and Bland-Altman analyses to quantify measurement error. The SEM indicates within-subject variability in repeated measures for a group of individuals [53]. The MDC_95_ is the smallest change necessary to exceed the measurement error of repeated measures that indicates a real change at the 95% confidence interval (CI) level for a single individual [53,54]. Bland-Altman analyses were used to indicate systematic bias between repeated measurements. The Bland-Altman plot illustrates the agreement between the 2 test occasions (time 1 and time 2) and identifies possible outliers. The 95% CI of the mean difference was used to determine systematic bias. If zero is included within the 95% CI, no significant systematic bias between measurements can be inferred. The 95% limit of agreement (LOA) was used to examine the natural variation over time, with a narrow LOA indicating higher stability [55,56].

## Results

A total of 123 subjects (43 males and 80 females) were enrolled in this study (Table 1). The average age was 46.12 years (range 20–87) for 121subjects due to 2 missing age information. The average total score of the MFI-TC at the first and second assessment was 47.28 and 46.85, respectively. The average total score of the SF-36-T was 597.86 and that of the PSQI was 5.81. The gender issue has been addressed in previous studies of the MFI [24,42] and showed primarily female participants. In this study, roughly twice as many females as males were included due to more female volunteers and more uncompleted questionnaire response by males. We compared the data of male and female participants and showed that there was no significant difference in age, education level, total score of the PSQI, and total score and subscales of the MFI-TC between male and female participants (*p* >0.05) except for the mental fatigue subscale score at retest *(p* = 0.049).

**Table 1:** Characteristics of the participants (*n =* 123).

Characteristic	All participants (n = 123)	Males (n = 43)	Females (n = 80)	*p*-value
Age ^a^	46.12 (18.40)	43.02 (16.99)	47.82 (19.02)	0.17
Gender				
Male	43 (35%)	43 (35%)		
Female	80 (65%)		80 (65%)	
Level of education ^a^				0.29
1–6 years	5 (4%)	1 (2%)	4 (5%)	
7–9 years	6 (5%)	0 (0%)	6 (8%)	
10–12 years	19 (15%)	6 (14%)	13 (18%)	
13–16 years	61 (50%)	25 (58%)	36 (49%)	
17 years or more	26 (21%)	11 (26%)	15 (20%)	
First assessment of the MFI-TC				
Total score	47.28 (13.65)	44.02 (13.92)	49.03 (13.26)	0.052
Subscales				
General/Physical Fatigue	18.10 (6.35)	16.67 (6.16)	18.86 (6.35)	0.07
Reduced Activity	12.33 (3.96)	11.47 (4.58)	12.79 (3.52)	0.08
Reduced Motivation	10.31 (3.76)	9.72 (3.74)	10.63 (3.76)	0.21
Mental Fatigue	6.54 (2.43)	6.16 (2.63)	6.75 (2.30)	0.20
Second assessment of the MFI-TC				
Total score	46.85 (14.41)	43.88 (14.09)	48.49 (14.25)	0.09
Subscales				
General/Physical Fatigue	17.58 (6.45)	16.42 (6.34)	18.20 (6.47)	0.15
Reduced Activity	12.12 (3.94)	11.37 (4.26)	12.53 (3.73)	0.12
Reduced Motivation	10.46 (3.82)	10.07 (4.00)	10.68 (3.73)	0.40
Mental Fatigue	6.68 (2.44)	6.09 (2.23)	7.00 (2.51)	0.049
SF-36-T total score	597.86 (139.66)	635.62 (124.85)	577.57 (143.67)	0.03
PSQI total score	5.81 (2.96)	5.30 (2.78)	6.09 (3.03)	0.16

Data are reported as number of participants (%), mean (SD). Abbreviation: SD, standard deviation; MFI-TC: Traditional Chinese version of the Multidimensional Fatigue Inventory; SF-36-T: Short-Form-36 Health Survey Taiwan Form (SF-36-T); PSQI: Pittsburgh Sleep Quality Index.

^a^: 2 subjects did not provide age information and 6 subjects did not provide education information.

### Construct validity of the formal MFI-TC

The results of the PCA are presented in Table 2. Four factors were extracted. The first factor was interpreted as a combination of the general fatigue and physical fatigue dimensions and the other 3 factors as the reduced activity, reduced motivation, and mental fatigue dimensions. All 20 items had a unique loading of >0.40 on 1 of the 4 factors in the pattern matrix. The Kaiser-Meyer-Olikin (KMO) value and Bartlett’s test of sphericity were used to gain an understanding of the appropriateness of factor extraction. Factor extraction was done by PCA, which involves varimax rotation and an eigenvalue larger than 1. The results showed that the KMO value was 0.89 (>0.80), and the result of Bartlett’s test was 1420.30 (*p*<0.001), which was suitable for factor analysis [57].

**Table 2:** Factor analysis of the MFI-TC.

Item	Traditional Chinese version		Factor loading	Variance (%)	Eigenvalue
Factor 1. General Fatigue/Physical Fatigue				41.99	8.40
1	我覺得我的體能很好。		0.68		
2	體力上我覺得我只能做一點點事情。		0.43		
5	我覺得我疲倦。		0.75		
12	我的休息是充分的。		0.76		
14	在體能上,我覺得我處在一個很糟的狀態。		0.56		
16	我很容易感到疲倦。		0.73		
20	在體能上,我覺得我處在一個很好的狀態。		0.65		
Factor 2. Reduced Activity				11.16	2.23
3	我覺得我很活躍。		0.71		
4	我覺得我想去做所有美好的事情。		0.54		
6	我認為我一天能做許多事情。		0.64		
8	我的體力可以讓我從事許多事情。		0.66		
15	我有許多的計畫。		0.75		
Factor 3. Reduced Motivation				7.00	1.40
9	我對要去處理事情感到畏懼。		0.68		
10	我認為我一天只能做一點點事情。		0.63		
17	我只能完成一點事情。		0.64		
18	我不喜歡做任何事情。		0.77		
19	我很容易恍神。		0.61		
Factor 4. Mental Fatigue				5.43	1.09
7	當我在做事情時,我能專心於那件事情上。		0.82		
11	我可以很專心。		0.70		
13	我需要很努力才能專心在事情上。		0.70		
Total variance (%)				65.58	
Kaiser-Meyer-Olkin (Bartlett’s Test of Sphericity)		0.89 (p < 0.001)			

MFI-TC: Traditional Chinese version of the Multidimensional Fatigue Inventory. MFI: MULTIDIMENSIONAL FATIGUE INVENTORY. Refer to Smets et al. (1995) and Elbers et al. (2012) for the original/ English text

Four factors were extracted, and the total variance explained was 65.58% (Table 2). This result was confirmed by a scree plot that demonstrated marked discontinuity after the fourth factor (S1 Fig). The 4 factors were: (1) general/physical fatigue, including 7 items that explained 41.99% of the variance; (2) reduced activity, which included 5 items that explained 11.16% of the variance; (3) reduced motivation, which included 5 items that explained 7.00% of the variance; and (4) mental fatigue, which included 3 items that explained 5.43% of the variance (Table 2).

Six items loaded on other factors compared to the original MFI subscales [22]. Item 8 (“Physically I can take on a lot”) loaded on factor 2 (reduced activity) instead of the physical fatigue subscale. Item 4 (“I feel like doing all sorts of nice things”) and item 15 (“I have a lot of plans”) loaded on factor 2 (reduced activity) instead of the reduced motivation subscale. Item 10 (“I think I do very little in a day”) and item 17 (“I get little done”) loaded on factor 3 (reduced motivation) instead of the reduced activity subscale. Item 19 (“My thoughts easily wander”) loaded on factor 3 (reduced motivation) instead of the mental fatigue subscale.

The factor loadings ranged from 0.43 to 0.82, representing the actual correlation between each item and the factor scores (Table 2). The factor correlation matrix shows fair to good correlations between factors (ρ = 0.36 to 0.68). Factor correlations between general/physical fatigue, reduced activity, reduced motivation, and mental fatigue and the MFI-TC total scores were 0.91, 0.81, 0.86, and 0.68, respectively (Table 3).

**Table 3:** Correlations between subscales and the total score of the MFI-TC.

	General/Physical Fatigue	Reduced Activity	Reduced Motivation	Mental Fatigue
MFI-TC Total score	**0.91**	**0.81**	**0.86**	**0.68**
MFI-TC subscales				
General/Physical Fatigue	-	**0.68**	**0.62**	**0.51**
Reduced Activity	**0.68**	-	**0.54**	0.36
Reduced Motivation	**0.62**	**0.54**	-	**0.63**
Mental Fatigue	**0.51**	0.36	**0.63**	-

Expressed as Spearman rho correlation coefficient. Absolute correlation coefficients of 0.5 to 0.75 are considered moderate-to-good correlation, in bold; > 0.75 are good-to-excellent correlation [43], in bold. MFI-TC: Traditional Chinese version of the Multidimensional Fatigue Inventory. All p-values are less than 0.0001.

### Convergent validity of the formal MFI-TC

The convergent validity of the MFI-TC was evaluated by calculating Spearman correlation coefficients between the SF-36-T and the MFI-TC and between the PSQI and the MFI-TC. The results showed that all the subscales and the total fatigue score of the MFI-TC were significantly and negatively correlated with the total score of the SF-36-T, ranging from -0.42 to -0.69 (Table 4). The correlation coefficients between the subscales of the MFI-TC and the 8 dimensions of the SF-36-T are shown in Table 4. The MFI-TC was significantly correlated with all 8 dimensions of the global quality-of-life scale of the SF-36-T. The MFI-TC total score correlated to a higher degree with the general health, vitality, and mental health domains than with the other domains of the SF-36-T (Spearman’s rho = -0.67 for general health, Spearman’s rho = -0.75 for vitality, and Spearman’s rho = -0.71 for mental health). It can be seen from Table 4 that general/physical fatigue of the MFI-TC, as well as reduced activity, also had significantly higher correlations with the general health, vitality, and mental health dimensions of the SF-36-T (Spearman's rho = -0.72, -0.75, and -0.69, respectively, for general/physical fatigue; Spearman’s rho = -0.57, -0.62, and -0.53, respectively, for reduced activity). In addition, the reduced motivation and mental fatigue domains of the MFI-TC were significantly correlated with each domain of the mental component of the SF-36-T (respectively, Spearman’s rho = -0.55 and -0.40 for vitality, -0.44 and -0.37 for social functioning, -0.37 and -0.45 for emotional roles, and -0.57 and -0.48 for mental health). All p-values are less than 0.0001 except for absolute correlations coefficient of less than 0.30.

**Table 4:** Correlations between fatigue, quality of life, and quality of sleep.

MFI-TC	SF-36-T									PSQI
	Physical component				Mental component				Total score	Total score
	Physical functioning	Physical roles	Bodily pain	General health	Vitality	Social functioning	Emotional roles	Mental health		
Total Fatigue Score	-0.33	-0.39	-0.26	**-0.67**	**-0.75**	**-0.55**	-0.45	**-0.71**	**-0.68**	0.47
General/Physical Fatigue	-0.37	-0.36	-0.28	**-0.72**	**-0.75**	**-0.55**	-0.42	**-0.69**	**-0.69**	0.46
Reduced Activity	-0.31	-0.29	-0.14	**-0.57**	**-0.62**	-0.38	-0.25	**-0.53**	**-0.52**	0.36
Reduced Motivation	-0.22	-0.29	-0.17	-0.47	**-0.55**	-0.44	-0.37	**-0.57**	**-0.50**	0.36
Mental Fatigue	-0.07	-0.30	-0.19	-0.33	-0.40	-0.37	-0.45	-0.48	-0.42	0.26

Expressed as Spearman rho correlation coefficient. Absolute correlation coefficients of <0.25 are considered low correlation; 0.25 to 0.5 are fair correlation; 0.5 to 0.75 are moderate-to-good correlation, in bold; > 0.75 are good-to-excellent correlation [43]. All p-values are less than 0.0001 except for absolute correlations coefficient of less than 0.30.

The MFI-TC total score and subscale scores were also significantly positively correlated with the PSQI, ranging from 0.26 to 0.47. All p-values are less than 0.0001 except for correlation between Mental Fatigue and PSQI Total score (Spearman’s rho = 0.004).

### Reliability of the MFI-TC

**Fig 1:**
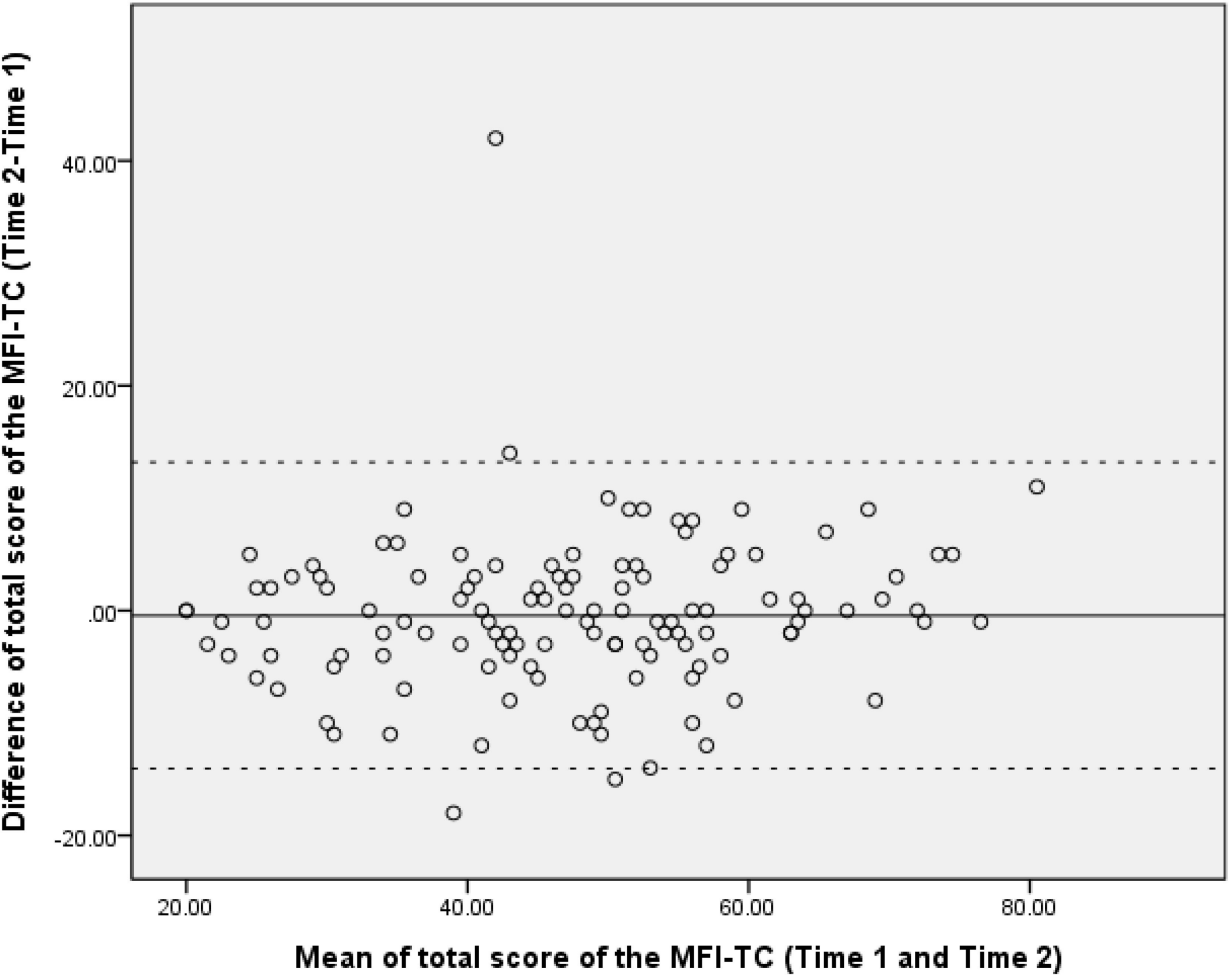
Bland-Altman plot of the total fatigue score of the MFI-TC. The plot illustrates the agreement between time 1 and time 2 and identifies possible outliers. Each subject is represented on the graph by conveying the mean value of the 2 assessments (x-axis) and the difference between the 2 assessments (y-axis). The mean difference was the estimated bias, and the standard deviation (SD) of the differences measured the fluctuations around this mean (outliers being above 1.96 SD_diff_). The reference lines show the mean difference between time 1 and time 2 (solid line), and the 95% limits of agreement for the mean difference (broken lines).

The Cronbach’s alpha coefficient was 0.92 for the total scale score of the MFI-TC, 0.89 for general/physical fatigue, 0.80 for reduced activity, 0.83 for reduced motivation, and 0.73 for mental fatigue (Table 5). Cronbach’s alpha coefficients indicated that the MFI-TC has a satisfactory internal consistency. The ICC rating was 0.94 for the total fatigue score, 0.93 for the general/physical fatigue subscale, 0.87 for the reduced activity subscale, 0.85 for the reduced motivation subscale, and 0.88 for the mental fatigue subscale. The SEM and MDC_95_ for the total fatigue score and subscales of the MFI-TC were within acceptable ranges (Total score: 3.43 and 9.50 points; general/physical fatigue: 1.69 and 4.68 points; reduced activity: 1.42 and 3.93 points; reduced motivation: 1.47 and 4.07 points; mental fatigue: 0.84 and 2.33 points). The Bland-Altman analyses showed no significant systematic bias between the repeated measurements. The four outliers were out of the 95% limits of agreement for the mean difference. A narrow range of the LOA was shown on the Bland-Altman plot, indicating the MFI-TC had high stability and low variation between the 2 test occasions (Fig 1).

**Table 5:** Reliability (internal consistency, test-retest, and measurement error) of the total score and four subscales of the MFI-TC in healthy adults (*n* = 123).

	Reliability		Measurement error		
MFI-TC	Internal consistency (Cronbach’s alpha)	Test-retest reliability ICC (95% CI)	SEM	MDC_95_	LOA
Total Fatigue Score	0.92	0.94 (0.91–0.95)	3.43	9.50	-14.05 to 13.19
General/Physical Fatigue	0.89	0.93 (0.90–0.95)	1.69	4.68	-7.01 to 5.97
Reduced Activity	0.80	0.87 (0.82–0.91)	1.42	3.93	-5.45 to 5.05
Reduced Motivation	0.83	0.85 (0.79–0.90)	1.47	4.07	-5.54 to 5.24
Mental Fatigue	0.73	0.88 (0.82–0.91)	0.84	2.33	-3.32 to 3.04

MFI, Multidimensional Fatigue Inventory; 95% CI, 95% confidence interval; ICC, intraclass correlation coefficient; SEM, standard error of measurement = SD_pooled_ × √(1 − ICC)], where SD_pooled_ is the standard deviation for all observations from test occasions 1 and 2; MDC_95_, minimal detectable change at the 95% CI level = 1.96 × √2 × SEM = 1.96 × √2 × SD_pooled_ × √(1 − ICC)], where 1.96 is the 2-tailed tabled z value for the 95% CI and √2 represents the variance of 2 measures; LOA, limits of agreement = d ± 1.96 SD_diff_, where d is the mean difference between the two test sessions (test session 2 minus test session 1) and SD_diff_ is the standard deviation of the mean difference.

## Discussion

This study provides evidence of the validity and test-retest reliability of the MFI-TC in quantifying fatigue in the general population. Factor analysis demonstrated that MFI-TC comprises 4 major factors, and the convergent validity of the MFI-TC was good. The reliability of the MFI-TC showed good test-retest reliability, with high agreement, small measurement error, and no systematic bias for the assessment of adult populations. These findings suggest that the results of the MFI-TC are valid and reliable for assessing fatigue in the general population.

With respect to construct validity, the results of factor analysis of the MFI-TC in the general population demonstrated the four-factor structure. General fatigue and physical fatigue could not be distinguished in the Traditional Chinese version of the MFI as one subscale measuring physical aspects of fatigue. A scree plot of the MFI-TC data from the general population showed that the Traditional Chinese version of the MFI retrieved 4 factors, the same as reported in patients with idiopathic Parkinson's disease [58], but different from the 5 factors reported in a US adult population [42] and the 3 factors reported in Chinese patients with cancer [27] and Polish-speaking patients with cancer [35]. The five-factor structure of the original MFI showed that factors were highly correlated and some items might have loaded on more than one factor [22]. The different number of factors found in this study was possibly related to differences in the translation of the items, study sample, and cultural experience. The translation of the items of general fatigue subscale loaded physical fatigue factor due to related physical fitness. General and physical aspects of fatigue have likewise not been separated. Therefore, when subjects rated their physical fitness on fatigue, people with low level of physical fitness were more associated with increased fatigue. That is why the items of general fatigue subscale had a significant tendency to assess physical fatigue in a Taiwanese population. The 5 factors in the study by Lin et al. were physical fatigue, mental fatigue, reduced activity, general fatigue, and reduced motivation [42]. These 5 factors explained 20.10%, 15.18%, 14.10%, 13.40%, and 6.87%, respectively, of the variance in the 20 items of the MFI. The PCA of the current study showed that the combined general/physical fatigue factor extracted 41.99% of the variance, which was more than 33.50% reported for the combined physical and general fatigue factors in the study by Lin et al. [42]. This finding may indicate that the MFI-TC has higher construct validity in a Taiwanese adult population than in a US adult population. The study participants in Lin et al. included not only well people, but also people with chronic fatigue syndrome-like illness with severe fatigue lasting more than 6 months and people with chronic unwellness with or without fatigue. This may explain the increased factorial complexity in the US adult population sample. Moreover, compared with the 4 factors in this study, only 3 factors of spiritual fatigue, mental fatigue, and physical fatigue were observed for the Simple Chinese version of the MFI [27] and 3 dimensions of physical fatigue, mental fatigue, and reduced motivation were found in the Polish version of the MFI [35]. Probable reasons for the divergent responses might be that some items focus on the experience of fatigue and other items are concerned with the consequences of fatigue. Fatigue items are not only related to tiredness, weakness, exhaustion of strength after physical exercise and activities, but also to weariness and perceived lack of energy. The adult Taiwanese population and patients with cancer seemed to interpret the questions differently. Patients with cancer may experience more psychological and mental fatigue than physical fatigue; while physical fatigue appears to have greater influence in the daily activities of the adult Taiwanese population.

Previous studies used SF-36 and PSQI as comparators to test correlation with fatigue measures [42,59,60]. Poor health-related quality of life is associated with greater fatigue in US adult populations with or without fatigue [42] and patients with coronary artery disease [60]. Fatigue is also associated with poor sleep in patients with multiple sclerosis [59], rheumatoid arthritis [14], and traumatic brain injury [15], and in spouse caregivers of cancer patients [16]. Our results found that convergent validity supported the theoretically anticipated relationship between fatigue (MFI-TC), quality of life (SF-36-T), and sleep disturbances (PSQI). The correlation coefficients of the MFI-TC subscales and the SF-36-T likewise supported convergent validity, which is consistent with results from previous studies [42,60].

In accordance with our expectations, total fatigue score of the MFI-TC, the PSQI, and total score of the SF-36-T were moderately correlated, indicating that fatigue, quality of life, and sleep disturbances are theoretically related concepts. Sleep disturbances were positively correlated with all 4 subscales of the MFI-TC. Quality of life was negatively correlated with all 4 subscales, especially the general/physical fatigue subscale. Although previous studies did suggest that fatigue was related to these variables, few studies have explored which dimension of fatigue is specifically associated with these variables [24,27,42]. The total fatigue score, the general/physical fatigue subscale, and the reduced activity subscale of the MFI-TC each had a significant correlation with the general health, vitality, and mental health dimensions in the SF-36-T. This confirms that the general/physical fatigue and reduced activity subscales represent both physical and psychological aspects of fatigue. Both reduced motivation and mental fatigue subscales of the MFI-TC were significantly correlated with mental component of the SF-36-T, which reflects the mental health concept of fatigue. Therefore, the findings from the current study add to our understanding of the relationship between specific dimensions of fatigue and related factors. In comparison with the SF-36-T and the PSQI, the MFI-TC was able to achieve good convergent validity.

Several potential physiological mechanisms might contribute to fatigue. Research has shown that sleep disturbance, depression, and inactive lifestyles contribute to fatigue [59,61,62]. In people with Parkinson’s disease, fatigue, mainly mental fatigue, had been characterized as one of the non-motor symptoms [63]. However, the results of factor analysis of our current study and previous studies [22,31,58] showed that physical fatigue was also an important component. A previous study using lab testing techniques revealed that physical fatigue had both peripheral and central fatigue components [64–66]. These findings suggest that, while the MFI is a broad tool for assessing the severity of fatigue, the physiological mechanisms of general/physical fatigue are still unclear. Future studies should seek to elucidate these mechanisms.

Among the 20 items of the MFI-TC, 10 items are stated positively and the other 10 items are stated negatively, which does not allow a set response and can be used to examine whether the respondents are carefully reading the questions or not. The high Cronbach’s alpha might therefore be the result of a tendency of respondents to agree with the given negative statements in the MFI-TC and disagree with the positive statements in the MFI-TC, which is taken to be indicative of much fatigue. In this study, the MFI-TC subscales exhibited adequate internal consistency, with Cronbach’s alpha coefficients ranging from 0.73 to 0.92, which agrees with results from previous studies [10,22,24,28,34,42,58]. Cronbach’s alpha for the subscales of the MFI-TC from a sample of Taiwanese adults in this study are in the same range as those reported for the adult German population [24] and for patients suffering from Chronic Fatigue Syndrome while receiving radiotherapy [22]. Compared with the results of a study by Smets *et al*. (1995) [22], our results showed that the alpha coefficients for the total score and the 4 subscales of the MFI-TC were greater than 0.70, indicating that the Traditional Chinese version of the MFI has good internal consistency. Thus, the MFI-TC is a tool that can be used to accurately assess the dimensions and severity of fatigue within a Chinese-speaking population and its level of interference with their daily activities.

Furthermore, the MFI is easy to administer. It has been used in many patient populations with satisfactory scalability and few missing responses. In this study, the MFI-TC showed high test-retest reliability over a 1-week interval, with ICCs ranging from 0.85 to 0.94 for the total fatigue score and the subscales, which is consistent with results from a previous study [58]. The readministration of the instrument after 1 week was a way to track the status of the subject on different dimensions of fatigue through time. The stability of the MFI-TC can be affected by the variation in the amount of change among participants’ fatigue level. Our findings demonstrate stable perceptions of fatigue among participants over a 1-week interval. This might be because at the beginning of the study, participants were asked not to change their physical status during the period of test and retest. A change in the subject’s condition may have resulted in changes in the perceived fatigue levels. Therefore, our findings demonstrated that the MFI-TC possesses high stability and low variation between the 2 test occasions.

Test-retest reliability is a measure of consistency of responses over time. A basic concept regarding test-retest reliability is the need to retest a stable population (i.e., retested participants must be in a stable condition over time) [67]. However, there is no evidence available to provide a better time period between questionnaire administrations for evaluating test-retest reliability of fatigue instruments. Studies of test-retest reliability for fatigue instruments have used varying intervals between test administrations. The interval has ranged from one week to six weeks [21,43,58,68]. To ensure fatigue measure without too many variables influencing the trait that naturally happen, we chose one-week interval for retesting. The time between the two test administrations may affect the test-retest reliability. An insufficient time period between test administrations might allow participants to remember their first answers, and the longer the interval the more change of variation of the construct to occur [69]. It is possible that the relatively short, one-week time interval of re-evaluation used in this study resulted in a potential for recall or carry-over effects to overestimate the test-retest reliability of the MFI-TC. Conversely, longer test-retest time period used in the previous study combined with the change status of the participants over time, which may have underestimated the reliability of the fatigue measure [68]. It was speculated that approximately 2 weeks might be an appropriate time interval for retesting [68,70]. However, the appropriate time interval should depend on the construct to be measured and the target population.

There are some limitations to interpreting the results of this study. The first is the representativeness of the sample used for the development of the MFI-TC. More females were included in this study and this might impact the generalizability of the results. However, due to the relative small number of sample size in the present study, further research will need to validate the gender issue.

The test-retest reliability of this study was based on a general population whose fatigue status was generally stable within 1 week. While applying this scale in populations with progressive diseases such as patients with Parkinson’s disease, the 1-week test-retest reliability might not be identical. Fatigue assessments using the MFI-TC in a sample of patient populations are needed. Another limitation was no other fatigue scales for convergent validity were used in this study. We chose quality of life (measured by SF-36-T) and sleep quality (measured by PSQI) as conceptual criteria to validate fatigue measures. The measures were correlated lowly with measures designed to quantify different but associated concepts and highly with measures of the same construct. Fair to moderate correlations between the total fatigue score of the MFI-TC and the total score of the SF-36-T and the PSQI as these scales may not be strongly related with fatigue assessment. Moreover, in this study, no cutoff point is defined for fatigue. Since there are no generally accepted cutoff scores in the literature, the prevalence of fatigue requires further investigation.

In conclusion, our study demonstrated that the Traditional Chinese version of the MFI has an appropriate construct validity, reasonable convergent validity, adequate internal consistency, high test-retest reliability, and low measurement error. Factor analysis found moderate subscale-total correlations, and high factor loadings also helped to clarify the psychometric meaning. Therefore, the MFI-TC is a reliable and valid instrument for comprehensively measuring fatigue in adult populations. The MFI-TC provides a unique set of subscales (general/physical fatigue, reduced activity, reduced motivation, and mental fatigue) designed to assess specific aspects of fatigue in the general population. It also offers an assessment tool for health professionals that can be used in patient populations. Evaluation of its psychometric properties in clinical populations is warranted before the MFI-TC can be used in clinical trials of fatigue.

## Supporting information

S1 Fig The scree plot of the MFI-TC data from the general population. The Traditional Chinese version of the MFI retrieved 4 factors.
(PDF)

S1 Table Reliability and concurrent validity of translation between original English MFI and modified MFI-TC version (n = 66). (PDF)

S1 Dataset All data of the MFI-TC, SF-36, and PSQI questionnaires (n = 123). (XLS)
