# Supplementary figures and images for: Validity and reliability of the Traditional Chinese version of the Multidimensional Fatigue Inventory in general population
Source: PLoS One. 2018 May 10;13(5):e0189850. doi: 10.1371/journal.pone.0189850 (PMC5945051; doi:10.1371/journal.pone.0189850)

Scree plot

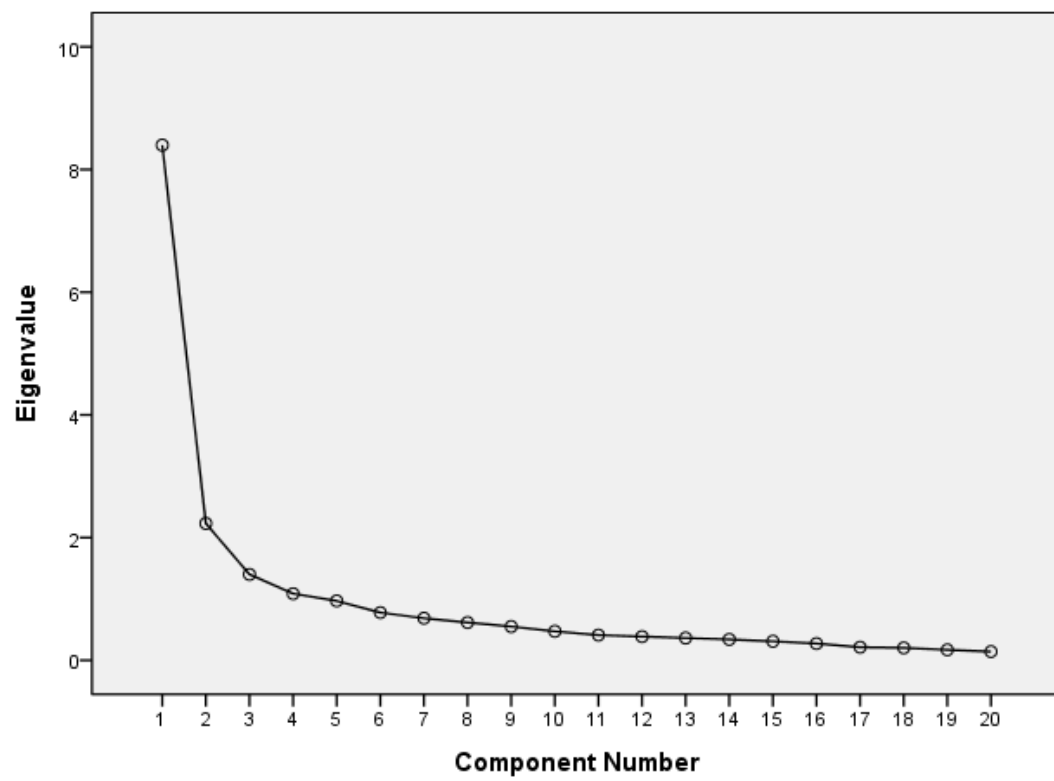

Supplement: S1 Fig — The Traditional Chinese version of the MFI retrieved 4 factors. (PDF) [file pone.0189850.s001.pdf]
