# Supplementary material for: Validity and reliability of the Traditional Chinese version of the Multidimensional Fatigue Inventory in general population
Source: PLoS One. 2018 May 10;13(5):e0189850. doi: 10.1371/journal.pone.0189850 (PMC5945051; doi:10.1371/journal.pone.0189850)
Supplement: S1 Table — (PDF) [file pone.0189850.s002.pdf]

MFI-TC	English version	Traditional Chinese version	ICC	Spearman rho correlation coefficient (ρ)
	mean (SD)	mean (SD)		
Total Fatigue Score	48.20 (11.86)	47.06 (11.97)	0.91	0.86
General Fatigue	11.35 (2.79)	10.92 (2.94)	0.90	0.81
Physical Fatigue	11.98 (1.10)	11.20 (1.50)	0.84	0.74
Mental Fatigue	9.33 (3.35)	9.01 (3.29)	0.92	0.83
Reduced Motivation	8.39 (2.41)	7.47 (2.44)	0.68	0.75
Reduced Activity	9.53 (3.00)	9.52 (3.11)	0.85	0.72

Note: ICC, intraclass correlation coefficient
